# In Vitro Investigation of a Terbinafine Impregnated Subcutaneous Implant for Veterinary Use

**DOI:** 10.1155/2012/436710

**Published:** 2012-07-19

**Authors:** M. J. Souza, T. Cairns, J. Yarbrogh, S. K. Cox

**Affiliations:** ^1^Department of Biomedical and Diagnostic Sciences, College of Veterinary Medicine University of Tennessee, Knoxville, TN 37996, USA; ^2^Melatek, LLC, 4738 Bergamot Way, Middleton, WI 53562, USA; ^3^Seimens Molecular Imaging, 810 Innovation Drive, Knoxville, TN 37932, USA

## Abstract

A terbinafine impregnated subcutaneous implant was evaluated to determine if drug was released into isotonic saline over the course of 6 months at two different temperatures, 37°C and 4°C. These temperatures were chosen to simulate the nonhibernating (37°C) and hibernating body (4°C) temperatures of little brown bats (*Myotis lucifugus*). Insectivorous bats of North America, including little brown bats, have been devastated by white nose syndrome, a fungal infection caused by *Geomyces destructans*. No treatments exist for bats infected with *G. destructans*. Implants were placed into isotonic saline; samples were collected once per week and analyzed with HPLC to determine terbinafine concentrations. The mean amount of terbinafine released weekly across the 28 weeks was approximately 1.7 **μ**g at 4°C and 4.3 **μ**g at 37°C. Although significant differences in the amount released did occur at some time points, these differences were not consistently greater or less at either of the temperatures. This study showed that terbinafine was released from an impregnated implant over the course of 6 months at concentrations ranging from 0.02 to 0.06 **μ**g/mL depending on temperature, which may be appropriate for little brown bats (*Myotis lucifugus*) infected with *Geomyces destructans*, the etiologic agent of white nose syndrome.

## 1. Introduction

Treatment of systemic fungal infections often requires from weeks to months of drug therapy. Consistently medicating companion animals for this length of time can be difficult and even more so with animals that become stressed with handling, such as wildlife or exotic pets. Nondomesticated animals are susceptible to stress from repeated handling and restraint, and stress can lead to the death of hospitalized wildlife or exotic pets [1]. Stress, including that associated with handling in animals, has also been shown to lead to immunosuppression and increased susceptibility to disease. Therefore, stress associated with repeated handling for treatment of an infection could inhibit an animal's ability to mount an appropriate immune response [2–4]. 

White nose syndrome, caused by the fungus *Geomyces destructans*, is an infection that affects insectivorous bats of North America [5–7]. Extremely high mortality rates are associated with infection and to date, no effective treatments have been found. If numbers of bats continue to decline and approach extinction, members of various affected species may need to be captured and kept in captivity in order to preserve genetic diversity for eventual release and repopulation. Animals may be infected with *G. destructans* when captured and need antifungal treatment that is long acting and which would require limited handling of the animal.

Terbinafine is a fungicidal medication that inhibits the synthesis of ergosterol which is an essential component of fungal walls. Although no studies examining this drug in bats have been published, studies have been performed in other animal species [8–12]. *Geomyces pannorum, *a fungus that is closely related to *G. destructans, *can cause infection in humans and is susceptible to terbinafine [13, 14]; no published reports regarding the sensitivity of *G. destructans* to terbinafine are available. Terbinafine has also been useful in other refractory mycotic infections in humans [15]. *Geomyces destructans* has been shown to be susceptible to other antifungal agents in vitro including fluconazole, but terbinafine has a better safety profile than many other commonly used antifungal medications [16–18].

The goal of this study was to investigate a terbinafine impregnated implant designed for subcutaneous placement over the dorsum of bats infected with *G. destructans*; the in vitro release of terbinafine from the implant was evaluated at two different temperatures, 4°C and 37°C, over the course of approximately 6 months. The two temperatures were chosen because they are similar to the body temperatures of hibernating (4°C) and nonhibernating (37°C) bats. This initial trial was designed to determine if terbinafine would release from the implant over the course of many months without degradation of the implant in an in vitro setting.

## 2. Materials and Methods

**Figure 1:**
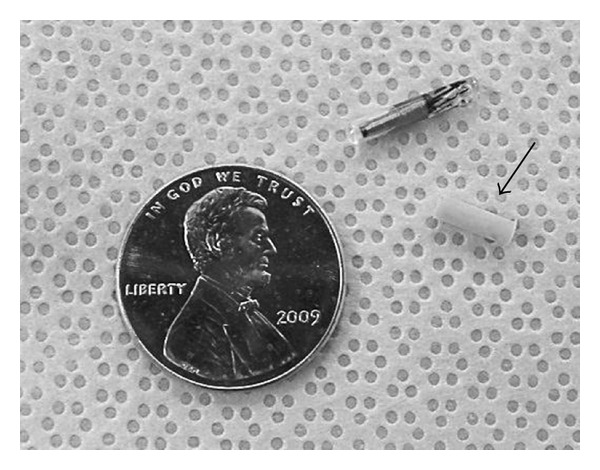
The terbinafine impregnated implant (arrow) is shown next to a PIT tag (microchip) and penny.

Implants were constructed by Melatek, LLC (Madison, WI, USA) based on protocols used to make Ferretonin implants. These implants are stable for approximately 5 years if kept at 4°C (T. Cairns, pers. comm.). Briefly, terbinafine HCl (Sigma-Aldrich, Co., St. Louis, MO, USA) was mixed with medical grade elastomer to a calculated concentration so that each implant would contain 0.5 mg of terbinafine. The mixture was placed into a mold where it cured and was then cut into individual implants. Each implant was approximately the size of a passive integrated transponder (PIT) tag (microchip) as shown in Figure 1. Cured medical grade elastomer is dimensionally and thermally stable, resistant to oxidation and sunlight, and does not become hard with age (T. Cairns, pers. comm.). Implants were kept at 4°C for approximately one month according to manufacturer instructions prior to placement into saline.

For in vitro analysis, implants were individually placed into 25 mL of isotonic saline in glass containers. Five implants were kept at 4°C and five were kept at 37°C. Every 7 days, the solution in each container was mixed to ensure homogeneity and 350 *μ*L was then removed and placed into a cryo-tube for analysis. Following sample removal, isotonic saline (350 *μ*L) was added to the container so the volume was kept consistent. Samples were collected for a total of 28 weeks and were kept in a −80°C freezer until analysis.

Saline samples were analyzed using HPLC with ultraviolet absorption. The system consisted of a 2695 separations module, a 2487 absorbance detector (Waters, Milford, MA, USA). Terbinafine was extracted from saline samples using a hexane extraction and was separated on a Symmetry Shield C_18_ (4.6 × 100 mm, 5 *μ*m) column with a guard column. The mobile phase was a mixture of (A) 20 mM phosphoric acid with 0.1% triethylamine adjusted to pH 3.0 and (B) acetonitrile (65 : 35). The flow rate was 1.1 mL/min and the column temperature ambient. Absorbance was measured at 224 nm.

Standard curves for analysis were prepared by fortifying saline with terbinafine to produce a linear concentration range of 5–1500 ng/mL. Average recovery for terbinafine was 95% while intra- and interassay variability were less than 10%. The lower limit of quantification was 5 ng/mL.

Following HPLC analysis, the amount of terbinafine released by each implant during each interval was calculated. The mean release of terbinafine with standard deviations was calculated for the different temperatures at each time point. Data was tested for normalcy with a Bartlett's test for inequality of variances. If the values were normally distributed, a *t*-test was performed to determine if a significant difference in amount of terbinafine released was present at the two temperatures. If the data was not normally distributed, a Mann-Whitney/Wilcoxon two-sample test was used to determine if differences existed. Significance was set at *P* < 0.05 and analysis was performed with EpiInfo (CDC, Atlanta, GA, USA).

## 3. Results

**Figure 2:**
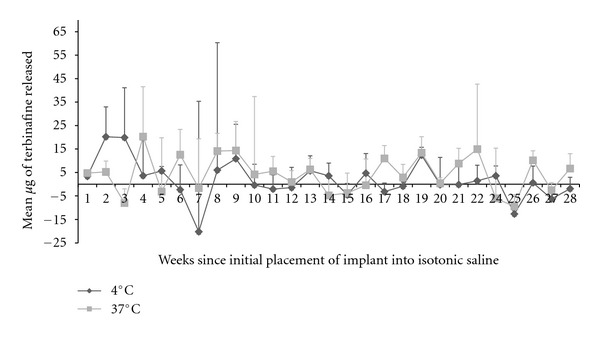
Terbinafine impregnated implants were placed into isotonic saline at 4°C (*n* = 5) and 37°C (*n* = 5). Samples were collected every 7 days and terbinafine concentrations were determined with HPLC. A sample was not collected during week 23. The mean amount (*μ*g) of terbinafine released (±SD) at the different temperatures is shown over the course of 6 months.

Samples were collected and analyzed with HPLC for a total of 28 weeks after initial placement into isotonic saline. A sample was not collected during week 23. The mean amount released from the implants at the two different temperatures during the 28 weeks is shown in Table 1/Figure 2. The amount released from the implants at 37°C was significantly greater than 4°C at the 1 (*P* < 0.01), 17 (*P* < 0.01), 26 (*P* = 0.03), and 28 (*P* = 0.04) week time points; the amount released from implants at 4°C was greater than 37°C at the 2 (*P* = 0.04) and 3 (*P* = 0.02) week time points. The mean amount of terbinafine released weekly across the 28 weeks was approximately 1.7 *μ*g at 4°C and 4.3 *μ*g at 37°C.

## 4. Discussion

The implant was evaluated at two different temperatures because of the differing rates of metabolism between hibernating and nonhibernating bats [19]. It was hypothesized that more terbinafine would be released at 37°C than at 4°C. If terbinafine was released from implants at different rates at the different temperatures, excessively high concentrations may be reached in nonhibernating bats or suboptimal concentrations may be reached in hibernating bats. In this study, there were significant differences between the release rates at 6 of the time points, but the levels were not consistently higher at one temperature compared to the other. Variations in the amount of drug released from the implants occurred at both temperatures and led to large standard deviations at some time points. This variation in drug release may have been due to slight differences in the temperature within the incubator/refrigerator or from incomplete mixing of the solution prior to sampling. Additionally, the measured concentrations at some time points indicated that the release was negative. These values may have been due to little if any release following the previous sample collection and replacement with saline which led to an overall lower concentration in the container. 

Terbinafine has been used in refractory fungal infections with success [15] and typically has fewer adverse effects than other antifungal medications [17]. Unpublished research has shown that *G. destructans* is susceptible to terbinafine, but minimum inhibitory concentrations (MIC) are not available. In vitro susceptibility of other fungi and yeasts ranges from 0.001 to 128.0 *μ*g/mL [17]. The mean amount of terbinafine released weekly during the 28 weeks was 1.7 *μ*g at 4°C and 4.3 *μ*g at 37°C. Assuming the typical little brown bat (*Myotis lucifugus*) weighs approximately 10 grams and this in vitro test is an appropriate approximation of the amount of terbinafine that would be released in vivo, bats would have a circulating concentration ranging from 0.02 to 0.06 *μ*g/mL for approximately 6 months depending on body temperature. These circulating concentrations would fall within the MIC for many pathogenic fungi and yeast, however, further studies are needed to determine the MIC of *G. destructans*. Additionally, initial clinical trials in little brown bats are currently being performed (M. Souza, pers. comm.). Implants were placed subcutaneously over the dorsum of bats infected with *G. destructans* and safety and efficacy of the implants will be determined. Results are not yet available, but skin samples will be evaluated with HPLC to determine terbinafine concentrations.

## 5. Conclusions

In conclusion, terbinafine was released from the implant over the course of 6 months with no consistent significant differences at two different temperatures, 37°C and 4°C. However, without in vivo absorption, metabolism and clearance, it is difficult to know whether this implant will release therapeutic amounts of terbinafine in *G. destructans* infected bats. This research was the first step to determine if terbinafine would release from the implant over an extended period of time and what amounts might be released. Future research will need to examine the implants in animals to determine the concentration of systemic terbinafine over time. Following further investigation, this implant may provide a long term treatment for *G. destructans* infected bats that requires handling only once at the beginning of treatment.

## Figures and Tables

**Table 1:** Mean (SD) amount of terbinafine (*μ*g) released from 0.5 mg implants in isotonic saline at two different temperatures, 4^°^C and 37^°^C. A *t*-test or Mann-Whitney/Wilcoxon 2-sample test was performed to determine if means differed between the two temperatures at the different time points. A *P* of <0.05 (*) denotes a significant difference.

Weeks after initial placement into saline	4^°^C		37^°^C		*P* value
	Mean	SD	Mean	SD	
1	3.4	0.6	4.8	0.2	<0.01^∗^
2	20.3	12.7	5.2	4.6	0.04^∗^
3	19.9	21.2	−8.0	6.1	0.02^∗^
4	3.7	15.1	20.3	21.3	0.19
5	5.7	1.9	−3.2	23.0	0.75
6	−2.3	10.6	12.6	10.8	0.06
7	−20.2	55.6	−1.6	21.0	0.70
8	6.0	54.3	14.2	7.5	0.17
9	10.9	14.7	14.4	12.4	0.70
10	−0.4	8.9	4.2	33.2	0.60
11	−2.1	6.0	5.5	6.3	0.09
12	−1.4	8.6	1.0	4.8	0.60
13	5.7	6.4	6.3	4.7	0.88
14	3.6	5.5	−4.7	9.3	0.12
15	−4.5	4.9	−3.7	8.5	0.86
16	4.7	8.4	−0.4	11.2	0.44
17	−3.2	3.7	11.0	5.5	<0.01^∗^
18	−0.8	1.8	2.9	5.6	0.19
19	12.4	3.3	13.4	6.9	0.78
20	−0.2	11.6	0.4	2.3	0.91
21	−0.1	8.8	8.8	6.5	0.10
22	1.5	6.6	15.0	27.7	0.32
24	3.6	4.2	−6.0	21.4	0.35
25	−12.7	7.5	−9.5	5.0	0.45
26	0.6	7.1	10.2	4.1	0.03^∗^
27	−6.3	2.5	−2.6	3.3	0.08
28	−1.9	4.9	6.7	6.4	0.04^∗^
